# Supplementary material for: Association between Endometriosis, Irritable Bowel Syndrome and Eating Disorders: ENDONUT Pilot Study
Source: J Clin Med. 2022 Sep 29;11(19):5773. doi: 10.3390/jcm11195773 (PMC9571159; doi:10.3390/jcm11195773)
Supplement: Supplementary file 1 [file jcm-11-05773-s001.zip › jcm-1919554-supplementary.pdf]

Supplemental data

S1-SCOFF QUESTIONNAIRE

positive if at least 2 items are answered positively

1/ Do you make yourself Sick because you feel uncomfortably full ?

YES

NO

2/ Do you worry that you have lost Control over how much you eat ?

YES

NO

3/ Have you recently lost more than One stone (14 lb) in a 3-month period ?

YES

NO

4/ Do you believe yourself to be Fat when others say you are too thin ?

YES

NO

5/ Would you say that Food dominates your life ?

YES

NO

S2-ROME IV QUESTIONNAIRE

positive if the first question is answered positively and at least 2 items from a, b or c

1/ Have you experienced recurrent abdominal pain, on average at least 1 day per week, in the past 3 months* ?

YES

NO

2/ Are these pains associated ?

a) To defecation

b) With a change in stool frequency

c) With a change in stool consistency

And rather with,

i) Constipation tendency

ii) Diarrhea tendency

iii) Alternating diarrhea and constipation

iv) Does not respond to the forms described

* with an onset of symptoms for at least 6 months

S3-EAT-26 QUESTIONNAIRE

positive if score ≥ 20

1/ I am terrified about being overweight :

Always (3)

Usually (2)

Often (1)

Sometimes (0)

Rarely (0)

Never (0)

2/ I avoid eating when I am hungry :

Always (3)

Usually (2)

Often (1)

Sometimes (0)

Rarely (0)

Never (0)

3/ I find myself preoccupied with food :

Always (3)

Usually (2)

Often (1)

Sometimes (0)

Rarely (0)

Never (0)

4/ I have gone on eating binges where I feel that I may not be able to stop :

Always (3)

Usually (2)

Often (1)

Sometimes (0)

Rarely (0)

Never (0)

5/ I cut my food into small pieces :

Always (3)

Usually (2)

Often (1)

Sometimes (0)

Rarely (0)

Never (0)

6/ I aware of the calorie content of foods that I eat :

Always (3)

Usually (2)

Often (1)

Sometimes (0)

Rarely (0)

Never (0)

7/ I particularly avoid food with a high carbohydrate content (i.e. bread, rice, potatoes, etc.) :

Always (3)

Usually (2)

Often (1)

Sometimes (0)

Rarely (0)

Never (0)

8/ I feel that others would prefer if I ate more :

Always (3)

Usually (2)

Often (1)

Sometimes (0)

Rarely (0)

Never (0)

9/ I vomit after I have eaten :

Always (3)

Usually (2)

Often (1)

Sometimes (0)

Rarely (0)

Never (0)

10/ I feel extremely guilty after eating :

Always (3)

Usually (2)

Often (1)

Sometimes (0)

Rarely (0)

Never (0)

11/ I am preoccupied with a desire to be thinner :

Always (3)

Usually (2)

Often (1)

Sometimes (0)

Rarely (0)

Never (0)

12/ I think about burning up calories when I exercise :

Always (3)

Usually (2)

Often (1)

Sometimes (0)

Rarely (0)

Never (0)

13/ Other people think that I am too thin :

Always (3)

Usually (2)

Often (1)

Sometimes (0)

Rarely (0)

Never (0)

14/ I am preoccupied with the thought of having fat on my body :

Always (3)

Usually (2)

Often (1)

Sometimes (0)

Rarely (0)

Never (0)

15/ I take longer than others to eat my meals :

Always (3)

Usually (2)

Often (1)

Sometimes (0)

Rarely (0)

Never (0)

16/ I avoid foods with sugar in them :

Always (3)

Usually (2)

Often (1)

Sometimes (0)

Rarely (0)

Never (0)

17/ I eat diet foods :

Always (3)

Usually (2)

Often (1)

Sometimes (0)

Rarely (0)

Never (0)

18/ I feel that food controls my life :

Always (3)

Usually (2)

Often (1)

Sometimes (0)

Rarely (0)

Never (0)

19/ I display self-control around food :

Always (3)

Usually (2)

Often (1)

Sometimes (0)

Rarely (0)

Never (0)

20/ I feel that others pressure me to eat :

Always (3)

Usually (2)

Often (1)

Sometimes (0)

Rarely (0)

Never (0)

21/ I give too much time and thought to food :

Always (3)

Usually (2)

Often (1)

Sometimes (0)

Rarely (0)

Never (0)

22/ I feel uncomfortable after eating sweets :

Always (3)

Usually (2)

Often (1)

Sometimes (0)

Rarely (0)

Never (0)

23/ I engage in dieting behavior :

Always (3)

Usually (2)

Often (1)

Sometimes (0)

Rarely (0)

Never (0)

24/ I like my stomach to be empty :

Always (3)

Usually (2)

Often (1)

Sometimes (0)

Rarely (0)

Never (0)

25/ I enjoy trying new rich foods. :

Always (0)

Usually (0)

Often (0)

Sometimes (1)

Rarely (2)

Never (3)

26/ I have the impulse to vomit after meals :

Always (3)

Usually (2)

Often (1)

Sometimes (0)

Rarely (0)

Never (0)

S4-FRANCIS SCORE - IRRITABLE BOWEL SYNDROME SEVERITY QUESTIONNAIRE

score from 0 to 500

1/ Are you currently suffering from abdominal pain ?

YES

NO

2/ If so, what is the intensity of the abdominal pain ?

0% 100%

No pain Mild pain Quite intense pain Severe pain Very severe pain

3/ Please indicate the number of days you suffer in a 10 day period ?

……………………. days

4/ Do you currently suffer from abdominal distension (bloating, swollen, tense stomach) ?

YES

NO

5/ If so, how serious are these abdominal distension problems ?

0% → 100%

No distension | Small distension | Fairly high distension | Significant distension | Very high distension

6/ How satisfied are you with your usual frequency of bowel movements ?

0% → 100%

Very satisfied | Satisfied | Fairly satisfied | Not satisfied | Not at all satisfied

7/ How does your irritable bowel syndrome affect or disrupt your life in general (answer this question only if you answered YES to questions 1 and 4) ?

0% → 100%

Not at all | Not much | Fairly | Totally

S5-HAD QUESTIONNAIRE

positive for anxiety if score on questions 1, 3, 5, 7, 9, 11, 13 ≥ 8

positive for depression if score on questions 2, 4, 6, 8, 10, 14 ≥ 8

1/ I feel tense or « wound up » :

Most of the time (3)

A lot of the time (2)

From time to time, occasionally (1)

Not at all (0)

2/ I still enjoy the things I used to enjoy :

Definitely as much (0)

Not quite so much (1)

Only a little (2)

Hardly at all (3)

3/ I get a sort of frightened feeling as if something awful is about to happen :

Very definitely and quite badly (3)

Yes, but not too badly (2)

A little, but it doesn't worry me (1)

Not at all (0)

4/ I can laugh and see the funny side of things :

As much as I always could (0)

Not quite so much now (1)

Definitely not so much now (2)

Not at all (3)

5/ Worrying thoughts go through my mind :

A great deal of the time (3)

A lot of the time (2)

From time to time, but not too often (1)

Only occasionally (0)

6/ I feel cheerful :

Not at all (3)

Not often (2)

Sometimes (1)

Most of the time (0)

7/ I can sit at ease and feel relaxed :

Definitely (0)

Usually (1)

Not often (2)

Not at all (3)

8/ I feel as if I am slowed down :

Nearly all the time (3)

Very often (2)

Sometimes (1)

Not at all (0)

9/ I get a sort of frightened feeling like « butterflies » in the stomach :

Not at all (0)

Occasionally (1)

Quite often (2)

Very often (3)

10/ I have lost interest in my appearance :

Definitely (3)

I don't take as much care as I should (2)

I may not take quite as much care (1)

I take just as much care as ever (0)

11/ I feel restless as I have to be on the move :

Very much indeed (3)

Quite a lot (2)

Not very much (1)

Not at all (0)

12/ I look forward with enjoyment to things :

As much as I ever did (0)

Rather less than I used to (1)

Definitely less than I used to (2)

Hardly at all (3)

13/ I get sudden feelings of panic :

Very often indeed (3)

Quite often (2)

Not very often (1)

Not at all (0)

14/ I can enjoy a good book or radio or TV program :

Often (0)

Sometimes (1)

Not often (2)

Very seldom (3)

S6-Anthropometry and Lifestyle Questionnaire

Name : …..................

First name : ….................

Present weight : …… kg

Height : …… m

Date of birth :

Marital status :

Single

Common-law union

Married

Divorced

Widow

What is your degree level ?

No diploma

Advanced level/High School Diploma

Bachelor's degree

Master's degree

Do you have a personal history of eating disorders ?

…………………………………………………………………………………………………
………

If so, which type ? (anorexia, anorexia-bulimia, bulimia, hyperphagia ?)

…………………………………………………………………………………………………
………

Do you have a personal history of irritable bowel syndrome ?

…………………………………………………………………………………………………
………

Do you have a personal history of psychiatric pathology ?

…………………………………………………………………………………………………
………

If so, which type ? (anxiety-depressive disorder ?)

………………………………………………………………………………………………………

………

When did you get your first menstruation ?

Before 12 years old

After 12 years old

What is the average length of time of your menstrual cycles ?

Less than 26 days

From 26 to 30 days

More than 30 days

Irregular

Stopped cycles

Have you ever had pregnancie(s) and if so, how many ?

………………………………………………………………………………………………………

………

How old were you when you are diagnosed with endometriosis ?

…… years old

1. BEFORE THE DIAGNOSIS OF ENDOMETRIOSIS :

Weight change? Weight loss? Weight gain?

………………………………………………………………………………………………………

……………………...

....

Before the diagnosis of your disease, for how many years had you suffered from symptoms (abdominal pain, digestive discomfort/disorders, transit disorders, irregular menstrual cycles,

bleeding outside of menstrual periods, fertility disorders, pain during sexual relations) ?

Less than 6 years

From 6 to 10 years

More than 10 years

Did you smoke cigarettes ?

Every day

More than 2 times a week

Less than 2 times a week

Never

Did you drink alcohol ?

Every day

More than 2 times a week

Less than 2 times a week

Never

Did you drink coffee ?

Every day

More than 2 times a week

Less than 2 times a week

Never

Did you eat fish ?

Every day

More than 2 times a week

Less than 2 times a week

Never

Did you eat red meat (beef, pork) ?

Every day

More than 2 times a week

Less than 2 times a week

Never

Did you eat white meat ?

Every day

More than 2 times a week

Less than 2 times a week

Never

Did you eat fruits and vegetables ?

Every day

More than 2 times a week

Less than 2 times a week

Never

Did you eat dairy products ?

Every day

More than 2 times a week

Less than 2 times a week

Never

Did you eat insdustrials products/in opposition to fresh products ?

Every day

More than 2 times a week

Less than 2 times a week

Never

Did you consume dietary supplements, vitamins ? If so, which one(s) ?

……

……

Did you practice physical activity ?

Never

Occasionally

Once a week

Several times a week

Every day

2. SINCE THE DIAGNOSIS OF ENDOMETRIOSIS :

Since your endometriosis diagnosis, how has your weight changed ? Have you lost weight ? Have you gained weight ?

…………………………………………………………………………………………………
…………………………………………………………………………………………………
…………………………………………………………………………………………………
…………………………………………………………………………………………………
………………………………

Since your endometriosis diagnosis, has your diet changed, and if so, what has changed ?

…………………………………………………………………………………………………
…………………………………………………………………………………………………
…………………………………………………………………………………………………
…………………………………………………………………………………………………
………………………………

Since your diagnosis of endometriosis, has your physical activity changed, and if so, how ?

…………………………………………………………………………………………………
…………………………………………………………………………………………………
………………

Do you have regular abdominal pain ?

…………………………………………………………………………………………………
………

If so, do they depend on your menstrual cycle or do they occur apart from your period ?

…………………………………………………………………………………………………
………

Are you on any anxiolytic medication ? If so, which one(s) and their posology ?

…………………………………………………………………………………………………
………

…………………………………………………………………………………………………
………

Do you have a contraceptive treatment ? If so, which one ?

…………………………………………………………………………………………………

…………………………………………………………………………………………………

…………..........

Are you on any treatments for your endometriosis (hormone treatments, painkillers)? If so, which one(s) and their posology ?

…………………………………………………………………………………………………
………

…………………………………………………………………………………………………
………
